# *Notes from the Field*: Multidisciplinary Approach to Investigating *Brucella canis* Exposures — South Carolina, September 2023

**DOI:** 10.15585/mmwr.mm7325a3

**Published:** 2024-06-27

**Authors:** Tori S. Moore, Ashlyn Lancaster, Julia Nelson, Jennifer Sanders, Marnie Johnson, Amanda Moore, Marco Tori

**Affiliations:** ^1^Epidemic Intelligence Service, CDC; ^2^South Carolina Department of Health and Environmental Control; ^3^Special Pathogens Laboratory, South Carolina Department of Health and Environmental Control; ^4^Division of State and Local Readiness, Office of Readiness and Response, CDC.

**Summary**

**What is already known about this topic?**

*Brucella canis* can be transmitted from dogs to humans through contaminated canine body fluids. No approved serologic tests for humans exist, making case identification challenging. Because cases are underreported, information on *B. canis* investigations is limited.

**What is added by this report?**

A pregnant stray dog exposed nine members of two households, eight veterinary clinic staff members, and five household pets before receiving a confirmed brucellosis diagnosis. A multidisciplinary approach to investigation and monitoring was implemented to identify exposures and recommend prophylaxis for humans. No secondary cases occurred.

**What are the implications for public health practice?**

Risk communication and testing of dogs clinically suspected to be infected with *B. canis* are critical for reducing spread of *B. canis* among dogs and to humans.

*Brucella canis*, a rarely diagnosed bacterial zoonotic organism that causes brucellosis in domestic and wild dogs, is considered an emerging zoonotic threat with worldwide distribution (*1*). *B. canis* infections in dogs and humans are likely underreported because symptoms are nonspecific, the organism is difficult to detect, and diagnostic tests vary widely in accuracy and reliability (*1*–*3*). Humans and animals can become infected through contact with contaminated canine body fluids and aborted materials. Symptoms in dogs generally include infertility and abortions; however, infected dogs might also be asymptomatic. Signs and symptoms in humans are nonspecific and include fever, joint pain, and fatigue; however, illness can be debilitating, including endocarditis, splenomegaly, or neurologic symptoms. No serologic tests for *B. canis* are approved for human diagnosis, making case identification challenging.

On September 27, 2023, South Carolina’s Department of Health and Environmental Control was notified by a local veterinarian that multiple persons had been exposed to a pregnant stray dog that had received a preliminary diagnosis of *B. canis* by indirect fluorescent antibody (IFA) testing after aborting her puppies. Epidemiologists and public health veterinarians investigated to confirm the animal’s diagnosis, identify additional human and animal exposures, and guide human treatment decisions. This activity was reviewed by CDC, deemed not research, and was conducted consistent with applicable federal law and CDC policy.*

## Investigation and Outcomes

On August 14, a stray dog wandered onto the property of a family of four that included two children and a dog. The family fostered the dog for 2 weeks, and on August 28, the dog was adopted by a family of five that included an infant and a toddler, as well as two dogs and two ferrets. On September 18, the dog was taken to a veterinarian after the owners observed vaginal discharge. The owners were informed the dog was pregnant; she aborted seven puppies on September 23, at which time the veterinarian submitted a *B. canis* IFA screening test. The dog was discharged the following day and kept isolated at home while awaiting results. On September 27, the *B. canis* IFA screening test result was reported as positive. 

Members of the foster and adoptive families and the veterinary clinic were interviewed by local epidemiologists to evaluate animal and human exposure risk. A total of 17 persons and five animals were exposed to the dog, including the foster family (and their dog), the adoptive family (and their two dogs and two ferrets), and eight veterinary clinic staff members.

On October 2, South Carolina’s Public Health Laboratory confirmed *B. canis* infection in the stray dog from culture and polymerase chain reaction testing of vaginal secretions; because of the poor prognosis (*2*) and the risk for zoonotic transmission, the dog was euthanized. The adoptive family’s two household dogs, that had contact with the infected dog while she was symptomatic, were screened using IFA testing; both received negative results. The adoptive family declined recommended 8-week follow-up testing for the household dogs (*2*). Three members of the adoptive family had directly handled aborted materials and puppies without personal protective equipment (PPE); because of these high-risk exposures, they received postexposure prophylaxis using a regimen extrapolated from existing brucellosis protocols^†^ and were monitored for symptoms for 24 weeks (*4*). All other exposed persons and animals, including the foster family and their dog, had lower-risk exposures, including collecting specimens while using PPE, feeding, petting, and walking the dog outside or, in the case of the ferrets, casual household contact. They were instructed to monitor for symptoms. At 24 weeks, no exposed persons reported symptoms in themselves or their pets.

## Preliminary Conclusions and Actions

This investigation, including collaboration between local veterinarians and pet owners, epidemiologists and public health veterinarians, physicians, and laboratorians, confirmed the diagnosis of *B. canis* in a stray dog and exposures of humans and pets. A multidisciplinary team of public health professionals collaborated to evaluate risk, direct animal testing, and recommend treatment. Since a serologic test for diagnosis in humans is not available, symptom monitoring for exposed persons and administering postexposure prophylaxis for persons with high-risk exposures was recommended.

Veterinarians should consider *B. canis* in a dog experiencing abortion or infertility. Testing should be performed to confirm clinical suspicion of *B. canis* in dogs (*2*). Molecular or rapid agglutination tests for dogs can assist in reducing spread of *B.* *canis* among dogs and guide treatment for exposed persons. Stray dogs or dogs housed in breeding kennels warrant a higher index of suspicion because of increased prevalence of brucellosis in these animal populations (*1*,*2*). Because no vaccine is available to prevent *B. canis* infection in dogs or humans, use of appropriate PPE by veterinary staff members examining dogs during delivery or dogs that are experiencing abortion is critical (*2*,*4*). Communication of the risk to pet owners and veterinary staff members is essential for reducing risk. Veterinarians should be familiar with the disease reporting requirements for brucellosis in their state or territory.

## Footnotes

* 45 C.F.R. part 46.102(l)(2), 21 C.F.R. part 56; 42 U.S.C. Sect. 241(d); 5 U.S.C. Sect. 552a; 44 U.S.C. Sect. 3501 et seq.

† Three weeks of doxycycline and rifampin.
